# Effect of power-law ionic conductances in the Hodgkin and Huxley model

**DOI:** 10.1186/1471-2202-16-S1-P250

**Published:** 2015-12-18

**Authors:** Fidel Santamaria

**Affiliations:** 1 Wodimu TekaUTSA Neurosciences Institute, University of Texas at San Antonio, San Antonio, TX 78249, USA

## 

An increasing number of results show that the voltage and spiking activity of a neuron follows scale free adaptation. Under such conditions, the voltage, or firing rate, of a neuron cannot be characterized by a unique time constant, instead these processes are characterized by power-laws. Power-law behaviors suggest that the components responsible for the voltage are strongly interacting across temporal scales, slow processes affect the rates of fast processes and vice versa. We recently introduced the fractional leaky integrate-and-fire model, which we have used to replicate the firing rate activity of adapting cortical neurons [1]. Our results have shown that spike rate adaptation can be modeled by a fractional derivative of low order. Thus suggesting, a strong interaction across conductances in cortical neurons.

this work we decided to study the effects of power-law behavior in a biophysical model of spiking activity, the Hodgkin-Huxley model. The classical Hodgkin-Huxley model is described by

$$C\frac{dV}{dt}=-\left(g_{m}\left(V-E_{rest}\right)+\overset{N}{\underset{i=1}{\sum }}g_{i}\left(V-E_{i}\right)\right)+I.$$

where C is the capacitance; V, voltage; g_m_, the membrane conductance; E_rest_, the resting potential; g_i_={g_Na_, g_K _} the sodium or potassium conductances with corresponding reversal potentials (E_i_={ E_Na_, E_K _}); and I, the input curret. The conductances are $g_{K}=\bar{g_{K}}n^{4}$ and $g_{Na}=\bar{g_{Na}}m^{3}h$, with $\bar{g_{K}}$ and $\bar{g_{Na}}$ the maximum conductances. The gating variables n, m, and h are defined by the general equation

$$\frac{dx}{dt}=\alpha_{x}\left(V,t\right)\left(1-x\right)-\beta_{x}\left(V,t\right)x$$

where x={n, m, h}. To implement power-law behavior in either gating variable we substitute the classical derivative by the fractional derivative of order η using the Caputo definition

$$\frac{d^{\eta }f}{dt^{\eta }}=\frac{1}{\Gamma \left(n-\eta \right)}\overset{t}{\underset{0}{\int }}\frac{f^{\left(n\right)}\left(t\right)}{{\left(t-u\right)}^{\eta +1-n}}du$$

where is the Gamma function, and n is the nearest integer larger than η. In our case, n = 1 because this models how the interaction of previous activity slows down the activation of the gating variables. The fractional derivative value is the result of integrating the activity of the function over all past activities weighted by a function that follows a power-law. The weighted values are called the memory trace. Thus, the fractional derivative provides information over all past activity, in contrast with the classical derivative that only takes into account the value of the function in the immediate previous time point. We have recently developed efficient ways to computationally solve these equations [2].

Our results show the emergence of a wide range of spiking behaviors (bursting, spiking and sub-threshold oscillations) in response to constant stimulation as a function of the fractional order in the different activation/inactivation variables. In the case of n, the neuron shows reduction if spiking response and emergence of sub-threshold oscillations. While fractional h results in bursting activity. This emergent richness in spiking activity, while only modeling two conductances, allows study of the overall effects of power-law behavior in neuronal activity. At the biophysical level, our results suggest that voltage dependent ion channels that deviate from a classical Markov process could increase the amount of spiking patterns in single cells, thus increasing their information content capacity.
